# Evaluation of Alternative Risk Stratification Systems in a Large Series of Solitary Fibrous Tumors with Molecular Findings and Ki-67 Index Data: Do They Improve Risk Assessment?

**DOI:** 10.3390/ijms24010439

**Published:** 2022-12-27

**Authors:** Isidro Machado, Álvaro Blázquez Bujeda, Francisco Giner, María Gema Nieto Morales, Julia Cruz, Javier Lavernia, Samuel Navarro, Antonio Ferrandez, Amparo Ruiz-Sauri, Antonio Llombart-Bosch

**Affiliations:** 1 Pathology Department, Instituto Valenciano de Oncología, 46009 Valencia, Spain; 2 Patologika Laboratory, Pathology Department, Hospital Quiron-Salud, 46009 Valencia, Spain; 3 School of Medicine, University of Valencia, 46009 Valencia, Spain; 4 Pathology Department, University Hospital “La Fe”, 46009 Valencia, Spain; 5 Zoology Department, University of Valencia, 46009 Valencia, Spain; 6 Department of Oncology, Instituto Valenciano de Oncología, 46009 Valencia, Spain; 7 Pathology Department, University of Valencia, 46009 Valencia, Spain

**Keywords:** solitary fibrous tumor, risk stratification system, Ki-67 index, *TP53* mutation, *HTER* mutation, overall survival

## Abstract

The clinical evolution of solitary fibrous tumors (SFTs) is often uncertain and several risk stratification systems (RSS) have been proposed. The Demicco et al. RSS is the most frequently implemented. In this study we aim to validate two alternative RSS (Sugita et al. and G-Score) using results for the Demicco RSS from a previous study of 97 SFTs. In addition, we aim to determine whether reclassified cases had any distinctive molecular features. As the Sugita et al. system substitutes mitotic count with Ki-67 index we also investigated whether Ki-67 results for tissue microarrays are comparable to those obtained using whole tissue sections. In the present study we detected that many cases classified by Demicco RSS as low-risk were reclassified as intermediate risk using the new system (G-score RSS). Kaplan-Meier survival plots for G-Score RSS showed that the low-risk and intermediate-risk SFTs had a similar evolution that contrasted with the more aggressive high-risk group. Moreover, the similar evolution in both low and intermediate-risk groups occurred despite the G-score system being stricter in classifying low-risk tumors. We observed that Sugita RSS does not provide any better risk stratification in comparison with the Demicco RSS, and testing both RSS in our series produced similar Kaplan-Meier survival data. We found some discordant results when comparing whole sections and the corresponding tissue microarrays samples, finding the hotspot areas easier to locate in whole sections. Forty-one SFTs with initial low-risk assigned by the Demicco RSS were reclassified as intermediate-risk by G-score finding both *TP53* and *HTER* mutations in four cases, only *HTER* mutation in 11 cases, and only *TP53* mutation in 2 cases. All six cases of SFT classified as high-risk by both the Demicco and G-score RSS suffered recurrence/metastasis, and half showed both *TP53* and *HTER* mutations. Five SFTs were categorized as low-risk by both Demicco and G-score, of which 4 cases revealed *HTER* mutation. Regarding the outcome of these 5 patients, two were lost to follow-up, and one of the remaining three patients suffered recurrence. We believe that although the presence of both *TP53* and *HTER* mutations may confer or be related to poor evolution, the isolated presence of *HTER* mutation alone would not necessarily be related to poor outcome. The G-score RSS more accurately identified low-risk patients than the other two risk models evaluated in the present series. Late recurrence/metastasis may occasionally be observed even in low-risk SFTs categorized by stricter classification systems such as the G-score RSS. These findings support the possibility that additional, as yet unknown factors may influence the clinical evolution of SFTs. In conclusion, given the possibility of late recurrence, long-term follow-up is recommended for all SFT patients, even in cases classified as low risk by the stricter G-score system. An integration of clinical, radiological, pathological, and molecular findings may improve SFT risk stratification and better predict patient outcome.

## 1. Introduction

Solitary fibrous tumors (SFTs) are mesenchymal neoplasms that can arise in any location, although they are infrequent in limbs [1,2,3,4,5,6,7,8,9,10,11,12,13,14,15,16,17,18,19,20,21,22,23,24,25,26,27,28,29]. The fusion gene *NAB2::STAT6* confirms a morphological diagnosis of SFT in cases with inconclusive STAT6 immunoreactivity [1,2,3,15,16,17,18,19,20,21,22,23,24,25,30,31,32,33,34,35,36,37,38,39,40,41,42,43]. Specific gene fusions have been related to prognosis and tumor location [1,6,8,13,26,27,28,29,30,31,32,33,34,38,41,42].

The clinical evolution of SFTs is often uncertain, and although most cases evolve in a benign fashion, a small group can progress towards recurrence and/or distant metastasis [1,2,3,4,5,6,7,8,9,10,11,12,13,14,15,16,17,18,19,20,21,22,23,24,25,26,27,28,29]. Recurrence rates of up to 30% in patients with localised SFT have been reported, and late recurrence may occur even in low-risk patients as much as 10 years after surgery [1,2,3,4,5,6,7,8,9,10,11,12].

Several risk stratification systems (RSS) have been proposed to predict recurrence in localised non-meningeal SFT [1,2,3,4,5,6,7,8,9,10,11,12]. The most implemented system is that of Demicco et al., which is based on mitotic count, age, tumor size, and necrosis [3,4,5]. Given that the evaluation of mitotic figures tends to differ between observers, Sugita et al. have developed a grading system that substitutes mitotic count with a Ki-67 index [11].

Furthermore, the identification of true low-risk patients due to poor prediction of late recurrence has been an inherent problem with these early RSS. Recently, Georgiesh T et al. [12] proposed a novel RSS (G-score) based on a large well-characterised patient cohort with long term follow-up. This new RSS includes mitotic count, necrosis, and gender as independent prognostic factors and is much stricter in its classification of low-risk SFTs [12]. While several RSS have been proposed and implemented, molecular results have not so far been included in any of the current RSS [3,4,5,6,7,8,9,10,11,12], despite the fact that they may provide additional prognostic information. For instance, Park HK et el. [27] have proposed that dysfunction of *TP53* and *APAF1* leads to reduced apoptotic function and eventually contributes toward malignant SFT transformation, something that may have significance in RSS.

We previously published a series of 97 SFTs in which the patients were classified using the Demicco et al. RSS plus additional molecular data (specific gene fusion as well as *p53* and *TERT* status) [10]. In this follow-up study we aim to validate the Sugita et al. [11] and G-score [12] RSS by comparing the results with the classification previously assigned in our series by the Demicco et al. RSS [4]. In addition, we aim to determine whether reclassified cases had any distinctive molecular features that might aid in the stratification of these tumors. Finally, to verify whether the Ki-67 results from tissue microarrays are comparable to those obtained with whole tissue sections.

## 2. Results

The clinicopathological and follow-up data as well as histologic, immunohistochemistry, and molecular data are provided in the previous study [10]. The median follow-up was 90 months.

### 2.1. Ki-67 in Whole Sections and Tissue Microarrays (TMAs) Sections

**Scheme 1:**
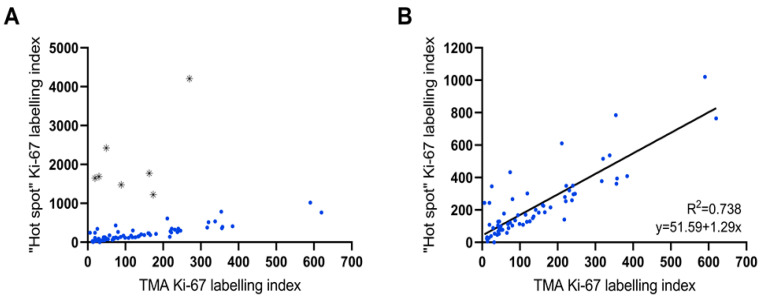
Correlation of Ki-67 labelling index of cell density (in mm^2^) between “hot spot” regions from whole section slides and TMA sections. (**A**) Dispersion graphic including all cases. 7 cases (shown as *) revealed a much greater positivity in the whole slide “hot spot” regions in comparison with the TMA sections and were considered outliers. (**B**) Simple linear regression of the data after censoring outliers. Line of identity, R2 and regression coefficients are shown. Abbreviations: TMA: Tissue microarray.

**Figure 1:**
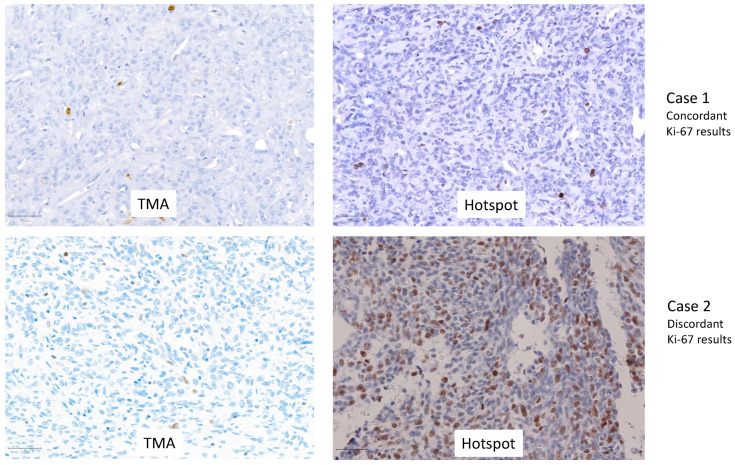
Ki-67 results in TMA and whole section (hotspot).

Ki-67 results are summarized in Scheme 1. Ki-67 results in whole section and TMAs revealed statistically significant differences with positivity being higher in the hotspot regions. As depicted in Scheme 1A,B, 7 tumors (9.46%) had higher positivity in the hotspot regions than in the TMA section. However, there was a good overall correlation between Ki-67 results in TMAs and hotspot regions from whole sections. Figure 1 show Ki-67 results in two cases.

### 2.2. Risk of Recurrence/Metastasis

**Scheme 2:**
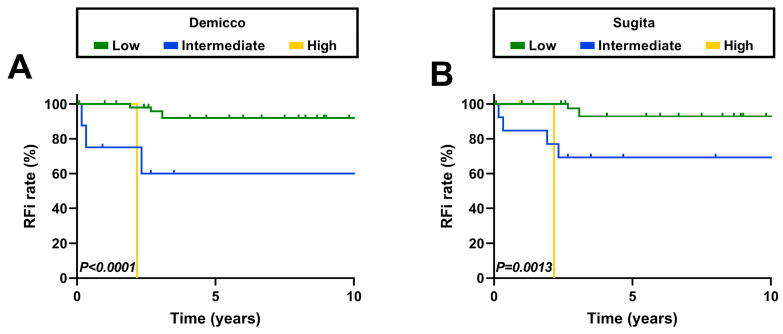
Prediction of recurrence risk using Demicco and Sugita et al., Kaplan–Meier survival curve analysis of recurrence-free interval including cases classified by using Demicco (**A**) and Sugita (**B**) RSS. Abbreviations: RSS: Risk stratification system.

**Scheme 3:**
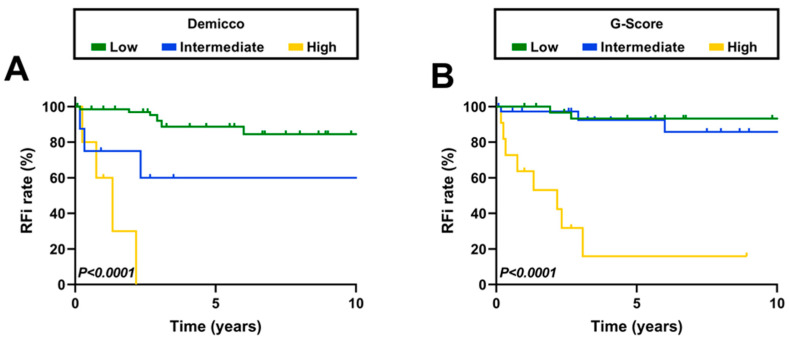
Prediction of recurrence risk using Demicco and G-Score. Kaplan–Meier survival curve analysis of recurrence-free interval including cases classified by using Demicco (**A**) and G-Score (**B**) RSS. Abbreviations: RSS: Risk stratification system.

The distribution of risk of recurrence and/or metastasis between the three RSS is described in Table 1. Table 2 summarizes the correlation between Demicco et al. and G-Score RSS for recurrence/metastasis, molecular alterations and outcome for all cases, showing both disagreement and agreement in assigned risk. Kaplan–Meier survival curve, including cases classified using Demicco and Sugita RSS are depicted in Scheme 2. Kaplan–Meier survival curve using Demicco and G-score RSS are shown in Scheme 3.

## 3. Discussion

Detection of the specific fusion gene *NAB2::STAT6* and its variants confirm a diagnosis of SFT, especially in infrequent clinical settings, uncommon histological findings or unexpected immunohistochemical profile [1,2,3,4,5,6,7,8,9,10,11,12,15,16,17,18,19,20,21,22,23,24,25]. In addition, specific gene fusions, *TP53* and/or *HTER* mutations have been related to prognosis [1,2,3,4,5,6,7,8,9,10,11,12,13,26,27,30,31,32,33,34], but have not so far been included in any risk stratification system [3,4,5,6,7,8,9,10,11,12].

Different risk-stratification systems (RSS) have been described [3,4,5,6,7,8,9,10,11,12], with the Demicco et al., system being the most widely implemented [4]. High-risk SFTs usually reveal recurrences and/or metastases and most of the low-risk SFTs categorized by Demicco et al. [4] and other RSS follow an apparently indolent course [3,4,5,6,7,8,9,10,11,12]. Nevertheless, some low-risk SFTs may have late recurrence/metastasis leading to uncertainty among clinicians regarding the specificity of the RSS [3,4,5,6,7,8,9,10,11,12,15,16,17,18,19,20,21,22,23,24,25,26,27,28,29]. In order to resolve this issue, a new RSS has recently been proposed (G-score), which is much stricter when stratifying a case as low-risk [12]. We observed in our series that many cases classified by Demicco RSS [4] as low-risk were changed to intermediate risk when classified using the new system (G-score RSS) [12], thus the total number of low-risk SFTs was reduced by using this new RSS. Kaplan-Meier survival plots in the present series using the Demicco system [4] showed three well-defined groups: low-risk, intermediate-risk and high-risk SFTs, this last being the group with poor evolution. However, using G-Score RSS [12], low-risk and intermediate-risk SFTs had a similar evolution that contrasted with the more aggressive high-risk group. Hence, although the G-score system [12] is much stricter when classifying tumors as low risk, the evolution for the low and intermediate-risk groups was similar, at least in the present series.

Among the histological predictive factors of aggressiveness in SFTs, high mitotic counts with a general agreement of ≥4/10 HPFs represent the strongest predictor of malignant behaviour [1,2,3,4,5,6,7,8,9,10,11,12,15,16,17,18,19,20,21,22,23,24,25,26,27,28,29,36,37,38,39,40]. Necrosis also represents an important histological factor, and both have been included in almost all RSS [1,2,3,4,5,6,7,8,9,10,11,12] including the latest G-score proposal [12]. However, although necrosis is apparently easy to assess by pathologists and radiologists, mitotic assessment may have some limitations, especially in limited samples or when mitotic figures may be overlooked due to tissue artifacts, difficulty in selecting the assessment area, necrosis with overlapping mitoses or abundant apoptotic figures [11]. In order to address these limitations, Sugita et al. recently published an RSS that replaces mitotic count with Ki-67 assessment [11]. Ki-67 assessment may offer some advantages compared with mitotic count, since for pathologists it is relatively easier to assess Ki-67 than mitoses. Nevertheless, we found that this RSS incorporating the Ki-67 index does not provide any better risk stratification in comparison with the Demicco RSS [4], and testing both RSS in our series produced similar Kaplan-Meier survival data. Intriguingly, in the present series, half the tumors categorized as low-risk by the Demicco et al. system [4] but which had a worse evolution (late recurrence or metastasis) showed Ki-67 ≥ 10. However, many of these cases were changed to intermediate risk using the new G-score RSS [12]. Finally, accurate and reliable Ki 67 index assessment requires whole tissue sections and digital pathology or morphometry methods. In the present series we observed some discordant results when comparing TMA samples and the corresponding whole sections, finding the hotspot areas easier to locate in whole sections.

Although clinical and histological parameters have been used to develop various RSS [3,4,5,6,7,8,9,10,11,12], molecular findings have not been included in any of the RSS in use so far. SFTs with *NAB2 exon 6-STAT6 exon 16/17* fusion occur in a significantly younger age group, showing higher mitotic activity and a higher recurrence rate [1,10,26,27,28,29,30,31,32,33,34,35,38,42]. We did not observe any direct association of gene fusion variants with aggressiveness or location of any histologic or phenotypic profile in the present series.

In the present study, we found 41 SFTs with initial low-risk assigned by the Demicco RSS [4] but which were reclassified as intermediate-risk when using the G-score [12]. Interestingly, we found both *TP53* and *HTER* mutations in four cases, only *HTER* mutation in 11 cases, and only *TP53* mutation in 2 cases. Both genes (*TP53* and *HTER*) have been associated with recurrence and malignant behaviour in SFT [1,2,3,4,5,6,7,8,9,10,11,12,26,27,28,29,30,31,32,33,34,35,36,37,38,39,40,41,42]. Out of the 41 patients with initial low risk assigned by Demicco RSS [4], 9 showed poor evolution with recurrence/metastasis and/or died of disease.

All six cases of SFT classified as high-risk by both the Demicco and G-score RSS [4,12] revealed recurrence/metastasis and half showed both *TP53* and *HTER* mutations. Five SFTs were categorized as low-risk by both Demicco and G-score [4,12], of which 4 cases revealed *HTER* mutation and 1 case had recurrence. We believe that although the presence of both *p53* and *HTER* mutations may confer or be related to poor evolution, the isolated presence of *HTER* mutation alone would not necessarily be related to poor outcome. These findings are in line with the previous observation of Demicco et al., where the *TERT* mutation probably provides no additional prognostic information on tumors already classified as low or high risk [26]. Furthermore, late recurrence/metastasis may occasionally be observed in SFTs categorized as low-risk by Demicco RSS [4] as well by other stricter systems as occurred with the G-score system [12] in the present series. These findings support the possibility that additional, as yet unknown factors may influence the clinical evolution of SFTs.

In this study, we classified all cases (*n* = 97) using the Demicco and G-score systems [4,12]. However, a limitation of the present study is that the Sugita et al., RSS [11] requires a Ki-67 score in both TMA sections and whole tissue which were not always available, therefore some cases were omitted from the evaluation of this system. Nevertheless, 60 cases were analysed using the Sugita et al. RSS [11] and the Kaplan-Meier survival plot revealed very similar results in comparison with the results obtained using the Demicco et al., RSS (*n* = 97). In addition, although we do not have the specific gene fusion in all cases in the present series, all cases showed strong and diffuse STAT6 nuclear expression by immunohistochemistry. This stain has been implemented in many laboratories as a very good surrogate for *NAB2::STAT6* gene fusion detection.

In conclusion, risk assessment remains a puzzling issue in SFT stratification and clinical outcome. Nevertheless, the integration of clinicopathological and molecular findings may improve risk stratification of SFTs and potentially may aid designing risk-adjusted treatment and scheduled follow-up. The addition of the Ki-67 index to RSS does not provide any prognostic impact and the survival curves are very similar to the plots obtained for the Demicco RSS [4], at least in the present series. Regardless of the assigned risk stratification score by any of the RSS, including G-score, SFTs may require long-term follow-up considering the fact that low-risk tumors may very occasionally show a more aggressive and unexpected evolution. We believe that the inclusion of molecular findings in RSS may improve precision in the stratification of SFTs, specifically, those SFTs with both *TP53* and *HTER* mutations which seem to evolve in a more aggressive fashion. Nevertheless, further studies are required to determine the most effective way to incorporate molecular analyses into RSS of SFTs. The G-score RSS more accurately identified low-risk patients than the other two risk models evaluated in the present series, although low-risk and intermediate-risk patients classified by the G-score system had a similar evolution. In conclusion, long term follow-up is recommended also in low-risk cases given the possibility of recurrence even in cases classified as low-risk by the stricter G-score system.

## 4. Materials and Methods

### 4.1. Patients and Samples

The study comprised 97 cases of histologically proven SFTs (STAT6 positive by immunohistochemistry). Formalin-fixed, paraffin-embedded tissue (FFPET) was retrieved from the archives at the Pathology Department, Clinical Hospital, University of Valencia; Hospital Universitari i Politécnic La Fe and Instituto Valenciano de Oncología (IVO) Valencia. The study was conducted in accordance with the principles of the Declaration of Helsinki and approved by the local Ethics Committee (IVO 2018-28). Clinical and follow-up data are described in the previous publication [10]. All the clinical data needed to stratify each patient according to RSS (age, gender and tumor size) [4,11,12] were retrieved from the previous study [10].

### 4.2. Assembly of Tissue Microarrays (TMAs)

Three tissue microarrays were performed using a manual tissue microarray instrument (Beecher Instruments, Sun Prairie, WI, USA). Each TMA comprised three cores (1 mm thick) of each sample. Following TMA construction, hematoxylin and eosin (H&E) stained section of each TMA was performed to confirm the presence of intact and representative neoplasm. Sections of 3 µm were cut in order to perform the immunohistochemical study.

### 4.3. Histopathology, Immunohistochemistry (IHC), and Molecular Analysis

All the histopathological data needed to stratify each patient according to the RSS (necrosis, mitotic count and tumor size) were retrieved from the previous study [10]. Immunohistochemistry staining for Ki-67 (Dako, clone MIB1, Ready-to-Use, low pH) was carried out on 3–4 µm-thick formalin-fixed paraffin-embedded tissue from a single representative block for each primary tumor section as well as for each tissue microarray. The reactions were detected using the EnVision system (Dako, Glostrup, Denmark). The extent of positive IHC reaction was scored as a percentage of nuclear positivity. All sections were evaluated independently and read in a blind manner by three pathologists (IM, FG, and ALLB). Discordant cases were evaluated at a multi-head microscope to achieve consensus. Standard positive and negative controls were used throughout. Any differences were resolved by agreement under multi-head microscope. Gene fusion, *p53,* and *TERT* molecular status were retrieved from the previous study [10].

### 4.4. Ki-67 Morphometric Quantification in Whole Sections and Tissue Microarrays

After immunohistochemical staining, the slides were scanned at a magnification of 20× using the VENTANA iScan HT slide scanner (Roche Diagnostics, Sant Cugat, Spain). The images obtained were then converted into OME-TIFF file format and imported into QuPath version 0.3.0 (Queen’s University, Belfast, Northern Ireland) for image analysis. Tissue microarray (TMA) slides were de-arrayed and pre-processed as previously described [43]. After de-arraying, TMAs were manually curated. The other slides were checked manually and cores of 1.2 mm diameter, equal to the ones generated with the de-arrayer in the TMA slides, were placed in the “hot spot” regions of each sample. In both types of slides, cell-detection was conducted using QuPath’s built-in ‘‘Positive cell detection’’ [43]. For each core, total detections, positive detections, negative detections and percentage of positive detections were assessed and exported into Microsoft Excel 365 (Microsoft, Redmond, WA, USA) for further analysis. The morphometric analysis was performed by (ABB and AR-S).

### 4.5. Risk of Recurrence/Metastasis

The risk of recurrence/metastasis was calculated using the Demicco scoring system [4], the Sugita et al. [11] RSS (in cases with informative Ki-67) and the G-score [12] RSS. The criteria for the three RSS are presented in supplementary Table S1.

### 4.6. Statistical Analysis

Survival analyses were performed in 96 patients with localized disease and available follow-up using the Kaplan–Meier method with log-rank tests and Cox proportional hazards models. Complete clinical and follow-up data are described in the previous study [10].

Survival was estimated by Kaplan–Meier curve analysis, and the log-rank (Mantel-Cox) test was run to determine if there were differences in the survival distribution between groups. For recurrence-free interval (RFI), distant metastasis or local recurrence was considered an event. The total duration of follow-up and time until occurrence of an event was calculated from the date of surgical resection or biopsy. Patients without recurrence were censored at the date of last radiological examination or last clinical follow-up.

Simple linear regression was used to test if microarray samples significantly predicted Ki-67 labelling index in “hot spot” regions, and Pearson correlations were computed to examine the intercorrelations between the two variables. Additionally, a paired Student’s t-test was performed to compare these two variables.

All statistical analyses were performed using SPSS Statistics, version 27.0 (SPSS Inc., Chicago, IL, USA) and GraphPad Prism, version 9.0.2 (GraphPad Software, San Diego, CA, USA). For all analyses, a *p* value < 0.05 was considered statistically significant.

## Figures and Tables

**Table 1:** Distribution of risk of recurrence and/or metastasis between the three RSS in the present series.

Risk Metastasis/Recurrence	Demicco et al. System	Sugita et al. System Cases with Ki-67 on Whole and TMA Sections	G-Score System
*Low*	80	47	41
*Intermediate*	11	11	44
*High*	6	2	12
**Total**	97	60	97

RSS: risk stratification system.

**Table 2:** Correlation between Demicco et al., and G-Score RSS for recurrence/metastasis, molecular alterations and outcome for all cases, showing both disagreement and agreement in assigned risk.

Molecular and Clinical Parameters	Low Risk by Demicco Intermediate Risk by G-Score N = 41	Low and/or Intermediate Risk by Demicco High Risk by G-Score N = 6	High Risk by Demicco High Risk by G-Score N = 6	Low Risk by Demicco Low Risk by G-Score N = 5
Only *p53* mutation	2	1	1	0
Only *HTER* mutation	11	2	1	4
*p53* and *HTER* mutations	4	0	3	0
Distant metastasis and/or local recurrence	4	5	6	1
Died of disease	5	5	3	0
Alive (free of disease)	34	1	0	2
Alive with disease	0	0	2	1
Lost to follow up	2	0	1	2

## Data Availability

Data is contained within the article or Supplementary Materials. In addition, any additional data presented in this study are available and would be requested to the corresponding author.

## Supplementary Materials

The following supporting information can be downloaded at: https://www.mdpi.com/article/10.3390/ijms24010439/s1, Table S1: The criteria for the three risk stratification systems.
