# Supplementary material for: Evaluation of Alternative Risk Stratification Systems in a Large Series of Solitary Fibrous Tumors with Molecular Findings and Ki-67 Index Data: Do They Improve Risk Assessment?
Source: Int J Mol Sci. 2022 Dec 27;24(1):439. doi: 10.3390/ijms24010439 (PMC9820154; doi:10.3390/ijms24010439)
Supplement: Supplementary file 1 [file ijms-24-00439-s001.zip › ijms-2077423-supplementary.pdf]

Supplementary Table S1

Prognostic factor	Demicco		Sugita		G-Score	
Mitoses (per 10 HPF)	Points<br>0<br>1<br>2	Value<br>0<br>1-3<br>≥4			Points<br>0<br>2	Value<br><4<br>≥4
Ki-67			Points<br>0<br>1<br>2	Value<br><1<br>1-10<br>≥10		
Age (years)	0<br>1	<55<br>≥55	0<br>1	<55<br>≥55		
Tumor size (cm)	0<br>1<br>2<br>3	0-4.9<br>5-9.9<br>10-14.9<br>≥5	0<br>1<br>2<br>3	0-4.9<br>5-9.9<br>10-14.9<br>≥5		
Necrosis	0<br>1	<10%<br>≥10%	0<br>1	<10%<br>≥10%	0<br>1<br>2	Absent<br><50%<br>≥50%
Gender					0<br>1	Female<br>Male
Scoring	Points<br>0-3<br>4-5<br>6-7	Risk<br>Low<br>Intermediate<br>High	Points<br>0-3<br>4-5<br>6-7	Risk<br>Low<br>Intermediate<br>High	Points<br>0<br>1-2<br>3-5	Risk<br>Low<br>Intermediate<br>High
